# Popliteus Tendon Morphology: Anatomical Classification and Clinical Implications—A Narrative Review

**DOI:** 10.3390/biomedicines13092053

**Published:** 2025-08-22

**Authors:** Łukasz Olewnik, Ingrid C. Landfald, Bartosz Gonera, George Triantafyllou, Daria Domosławska, Maria Piagkou, Robert F. LaPrade

**Affiliations:** 1 Department of Clinical Anatomy, Mazovian Academy, 09-402 Płock, Poland; ingridceciliee@gmail.com (I.C.L.); b.gonera@mazowiecka.edu.pl (B.G.); d.domoslawska@mazowiecka.edu.pl (D.D.); 2 Department of Anatomy, Faculty of Medicine, National and Kapodistrian University of Athens, 11527 Athens, Greece; georgerose406@gmail.com (G.T.); piagkoumara@gmail.com (M.P.); 3 Twin Cities Orthopedics, Edina, MN 55435, USA; laprademdphd@gmail.com

**Keywords:** popliteus tendon, posterolateral corner, knee stability, anatomical variation, ACL reconstruction, rotational instability, diagnostic imaging, posterolateral knee surgery, sports orthopedics, knee rehabilitation, posterolateral drawer test, dial test

## Abstract

**Purpose**: The popliteus tendon (PT), though often overlooked, plays a vital role in the functional and mechanical stability of the posterolateral corner (PLC) of the knee. This narrative review consolidates the current anatomical, biomechanical, imaging, clinical, and surgical data on the PT, with an emphasis on its morphological variability and relevance in orthopedic sports medicine. **Methods**: A comprehensive review of the literature was conducted, including classical anatomical studies, recent classification systems, biomechanical evaluations, imaging protocols, and rehabilitation strategies. Particular focus was given to the anatomical classification proposed by Olewnik et al. and its implications in surgical and diagnostic contexts. **Results**: Anatomical investigations have demonstrated considerable variability in the PT, including bifid tendons and accessory fascicles. These variants have a measurable impact on preoperative planning, diagnostic imaging interpretation, and outcomes of surgical procedures, such as anterior cruciate ligament (ACL) and PLC reconstructions. The PT also contributes significantly to knee rotational control and meniscal stabilization, particularly in athletic populations. Imaging modalities, such as MRI and dynamic ultrasound, show high diagnostic utility, while arthroscopy remains the definitive diagnostic and therapeutic modality. Rehabilitation should emphasize neuromuscular re-education and progressive control of tibial rotation. A phase-based rehabilitation framework and clinical action table are proposed. **Conclusions**: The PT should be recognized as a critical structure in both the conservative and the surgical management of posterolateral and rotational knee instability. Enhanced awareness of its anatomical variability and functional importance can improve diagnostic accuracy, surgical precision, and clinical outcomes. In particular, MRI and high-resolution ultrasound can aid in identifying accessory fascicles and bifid tendons, while arthroscopy benefits from preoperative knowledge of PT variants to avoid misidentification and iatrogenic injury. Surgical planning for ACL and PLC reconstructions may be refined by applying the classification system described. Future research should focus on refining diagnostic algorithms, developing PT-specific functional tests, and integrating popliteus evaluation into high-level clinical decision-making and surgical navigation systems.

## 1. Introduction

The popliteus tendon (PT), though relatively small in size, plays a disproportionately significant role in knee joint stability and function [1,2]. Anatomically, it forms part of the posterolateral corner (PLC) of the knee and contributes to both dynamic and static stabilization, particularly against external tibial rotation, varus stress, and posterior translation [3,4]. Injuries involving the PT are often underdiagnosed and frequently overlooked in clinical practice, yet they may contribute to persistent instability or chronic lateral knee pain when not properly addressed [5].

In the context of PLC instability, renewed focus on the PT has emerged due to its critical role in rotational control and its biomechanical interdependence with the cruciate ligaments [6,7]. Recent biomechanical models, including inverse dynamic simulations and robotic studies, have shown that dysfunction or injury to the PT significantly alters knee kinematics, particularly in loaded flexion or pivoting maneuvers [8,9]. Notably, the PT has also been identified as an essential component of the neuromuscular sensorimotor loop, participating in proprioceptive feedback, and modulating fine rotational control [10].

Recent anatomical studies have highlighted the morphological complexity and variability of the PT, suggesting a more nuanced understanding of its insertions, fiber architecture, and associated fascial connections [11,12]. Cadaveric investigations have revealed multiple configurations of PT insertions, accessory fascicles, and even direct capsular attachments, which may have implications for diagnostic imaging, surgical planning, and rehabilitation strategies [13,14]. The anatomical variability observed across individuals also raises questions regarding the role of congenital differences versus acquired adaptations in athletes and postoperative patients [15,16].

Despite the growing interest in the PLC as a whole, the literature specifically focusing on the PT remains limited and scattered. This presents challenges for clinicians who require detailed anatomical and biomechanical insight to inform personalized treatment, especially in complex cases, such as multiligamentous trauma, subtle rotational instability, or revision surgery after failed ligament reconstruction [6,7].

Given the heterogeneity and fragmentary nature of the available studies, ranging from anatomical dissections to biomechanical simulations and isolated clinical reports, a narrative review format was chosen to allow for integrated synthesis and clinically applicable interpretation.

The purpose of this review is to consolidate and to critically analyze the current anatomical, biomechanical, radiological, and clinical knowledge concerning the PT. Special emphasis is placed on morphological variability, diagnostic considerations, and the implications of such variability for surgical and conservative treatment approaches [1,17]. By synthesizing the available evidence, this review aims to serve as a foundational reference for orthopedic surgeons, radiologists, and sports medicine specialists interested in optimizing diagnosis and intervention strategies for injuries involving the PT [18,19].

## 2. Anatomy and Morphological Variability of the Popliteus Muscle

### 2.1. Normal Anatomy of the Popliteus Muscle

The popliteus muscle (PPM) is a small, triangular shaped deep muscle situated in the posteromedial aspect of the knee. It originates via a strong tendinous attachment from the lateral femoral condyle, near the origin of the fibular collateral ligament (FCL) and the posterolateral joint capsule [11]. The muscle belly runs obliquely in an inferomedial direction, inserting onto the posterior surface of the tibial shaft, just above the soleal line [2].

Functionally, the PPM plays a critical role in knee biomechanics. It initiates flexion by “unlocking” the extended knee achieved either through internal rotation of the tibia relative to the femur or, in weight bearing, by externally rotating the femur on a fixed tibia. Additionally, the muscle contributes to the stabilization of the lateral meniscus and reinforces the PLC by limiting excessive external tibial rotation and posterior tibial translation [1,18].

The PPM is innervated by a branch of the tibial nerve (L4–S1), which enters the muscle from a posterior-medial aspect. Its arterial supply arises primarily from the popliteal artery, with contributions from the superior lateral and inferior medial genicular arteries [15].

Anatomically, the PT courses deep to the FCL and closely approximates the lateral meniscus and the joint capsule. Its femoral attachment is distinct and separate from adjacent ligamentous insertions. The muscular belly, with its fan-shaped morphology, spans a broad area of the posterior tibial cortex, enabling efficient force transmission and dynamic stabilization [3].

Accurate knowledge of the normal anatomy of the popliteus and its spatial relationships is vital for both diagnostic imaging and surgical procedures involving the posterior aspect of the knee. Familiarity with the course of the tendon and its proximity to surrounding structures is particularly important in arthroscopic approaches and open PLC reconstructions [2,7].

### 2.2. Anatomical Variations

#### 2.2.1. Historical Perspectives on the Anatomy of the Popliteus Muscle

The anatomical understanding of the PPM has its foundations in the detailed descriptions of several prominent anatomists of the 19th century. Their classical observations continue to underpin modern interpretations of this muscle’s structure and clinical relevance. In his seminal work *Descriptive and Surgical Anatomy* (1875), Alexander MacAlister provided one of the earliest systematic descriptions of the PPM. He characterized it as a small, triangular muscle located on the posterior aspect of the knee, originating from the lateral femoral condyle and inserting on the posterior surface of the tibia above the soleal line. MacAlister emphasized its role in initiating knee flexion by internally rotating the tibia relative to the femur, a mechanism crucial for “unlocking” the extended knee [20]. Léo Testut, in his authoritative treatise, *Traité d’anatomie humaine* (1895), elaborated on the functional anatomy of the popliteus. He confirmed the muscle’s origin and insertion points, and reinforced its function in internal tibial rotation and posterior joint stabilization [21]. Testut’s accounts enriched the anatomical lexicon by situating the popliteus within the broader context of dynamic knee mechanics [22]. Wenzel Gruber [23], by contrast, focused on anatomical variability. His cadaveric studies revealed that the popliteus may exhibit accessory fascicles and variations in its points of attachment. These findings anticipated the clinical relevance of popliteus variants, particularly in the context of posterolateral knee injuries and surgical reconstructions [24]. Louis LeDouble extended this line of inquiry in his 1897 monograph, *Traité des Variations du système musculaire de l’homme*, where he systematically cataloged the deviations in muscular anatomy across populations. Regarding the popliteus, LeDouble reported the presence of supernumerary heads and atypical tendon trajectories. He suggested that such anatomical diversity might influence joint function and surgical outcomes [25].

These foundational contributions have served as a reference point for contemporary anatomical studies, which continue to explore the structural complexity and variability of the PPM with advanced imaging and dissection techniques.

#### 2.2.2. Recent Studies on the Morphological Variability of the Popliteus Muscle

Despite the biomechanical importance of the PPM, particularly within the posterolateral aspect of the knee, the presence and classification of its accessory bands remain poorly understood. To date, no universally accepted classification system exists to assist surgeons in preoperative planning involving the PT and its surrounding structures [1,15].

Previous anatomical investigations have largely focused on describing the spatial relationship between the FCL and the PT insertion, offering limited insight into the broader morphological spectrum of the PPM [1,3]. Moreover, several studies have attempted to identify and to classify variations in the proximal attachment of the PT, but these were often based on small cadaveric samples and lacked systematic standardization [7,15,19].

Feipel et al. [15] contributed to this area by outlining various possible attachment sites of the PPM tendon, which included the posterior joint capsule, the arcuate popliteal ligament, the oblique popliteal ligament, the fibular head, the lateral meniscus, the posterior cruciate ligament, and the posterior meniscofemoral ligament. Jung et al. [16] proposed a three type classification system based on the relationship between the PT and the FCL, including (1) the posteroinferior, (2) the directly inferior, and (3) the bifurcated tendon with dual insertions.

A significant step forward in the anatomical understanding of the popliteus muscle-tendon complex was made by Olewnik et al. [11], who in 2021 presented the first systematic and detailed morphological classification of the PT. Based on a study of 134 lower limbs, their classification considered the number of tendinous elements within the popliteal sulcus and the presence and configuration of accessory bands, offering a new anatomical framework with potential surgical relevance.

Their classification system divides the PLT into four main types (Type I–IV), with Type II and IV further subdivided based on the number and location of accessory bands. This approach allows for a more accurate recognition of anatomical variants that may impact surgical approaches to the PLC of the knee.

The specific types and subtypes described by Olewnik et al. [11] are summarized in Table 1.

#### 2.2.3. Clinical Implications of the Popliteus Tendon Classification

The detailed classification of the PT morphology proposed by Olewnik et al. [11] has substantial clinical significance, particularly in the context of posterolateral knee surgery, arthroscopic procedures, and anatomical graft harvesting. Recognition of the structural variability of the PT and its accessory bands is essential for avoiding iatrogenic injury, ensuring complete anatomical restoration, and improving surgical precision [1,17].

From a surgical perspective, Type I and Type III configurations, both characterized by isolated tendon insertions without accessory bands, may allow for more predictable intraoperative dissection and exposure [3]. Conversely, Type II and Type IV configurations, which involve one or more accessory bands inserting into key stabilizing structures (e.g., the FCL, the oblique popliteal ligament, the lateral meniscus, and the posterior capsule), may present a higher risk of intraoperative misidentification, especially during minimally invasive or arthroscopic approaches [6,15].

In particular, accessory bands attaching to the posterior horn of the lateral meniscus (Types IIc, IVc, IVd, IVe) may lead to diagnostic confusion during meniscal repairs or partial meniscectomies, potentially being misinterpreted as pathological adhesions or fibrous bands [18,19]. Similarly, accessory fibers inserting into the FCL or the posterior capsule may influence posterolateral stability, and thus should be preserved or anatomically reconstructed in cases of PLC injury [4,7,26].

Moreover, the presence of bifurcated or multiple tendinous components, as seen in Types III and IV, should prompt surgeons to re-evaluate assumptions about the “standard” tendon course during arthroscopic visualization [2]. Misidentifying a bifurcated PT for torn tissue may lead to unnecessary debridement or incomplete repair.

Understanding the full spectrum of anatomical variants not only facilitates more personalized surgical planning, but may also enhance outcomes in PLC reconstructions, revision ACL surgeries, and complex meniscal procedures [6,17]. Incorporating preoperative imaging (MRI or high-resolution ultrasound) focused on the popliteus region could improve identification of these variations and allow for safer, anatomy preserving techniques [1,3].

## 3. Biomechanics of the Popliteus Tendon

The PT is a key dynamic stabilizer of the knee joint, particularly involved in the control of rotational and posterior tibial motion. While small in size, it plays a disproportionately important biomechanical role in both static and functional knee stability. Its relevance is particularly heightened in situations involving PLC injury, cruciate ligament deficiency, and rotational instability [1,19].

### 3.1. Primary Functions of the Popliteus Tendon

Biomechanically, the PT functions as follows:

- To internally rotate the tibia (or externally rotate the femur in weight bearing),
- To unlock the knee from full extension by initiating flexion,
- To stabilize the lateral meniscus during knee motion,
- To resist external tibial rotation and posterior tibial translation, particularly in flexion beyond 30° [3,15].

As part of the PLC, the PT acts synergistically with the FCL and the popliteofibular ligament (PFL) to resist varus stress and posterolateral instability. Its action becomes especially critical during early stance and pivoting movements, when rotational control is required [4].

### 3.2. Cadaveric and In Vitro Evidence

Few biomechanical studies have demonstrated the crucial role of the PT in stabilizing the knee against external rotation. Gollehon et al. [27], in a landmark cadaveric study, showed that isolated sectioning of the PT increased external tibial rotation by approximately 7° at 30° of knee flexion. When combined with sectioning of the FCL and the PFL, the rotational instability rose dramatically, confirming the interdependence of the PLC components.

LaPrade et al. [1] further quantified these effects by demonstrating that PT resection increased the tibial external rotation and posterior translation by 2–4 mm in knees flexed beyond 45°, with the greatest effect observed during the pivot–shift maneuver. Their work confirmed that the PT is a major contributor to resisting tibial motion in both sagittal and transverse planes [1].

### 3.3. Role in Cruciate Ligament Deficient Knees

In ACL or PCL deficient knees, the PT plays a compensatory role. It provides secondary restraint to posterior translation in PCL deficient knees and augments control of rotational instability in ACL deficient knees [3,18]. If unrecognized and left untreated during cruciate ligament reconstruction, residual laxity may persist despite technically successful cruciate ligament graft placement.

Studies by Musahl et al. [9] and Bernhardson et al. [28,29] demonstrated that failure to reconstruct the PLC in multiligament injured knees, especially without restoration of the popliteus function, led to graft overload and failure. Consequently, preservation or reconstruction of the PT is increasingly emphasized during revision ligament surgery.

### 3.4. Dynamic Function in Gait and Athletic Movements

Electromyographic studies have shown that the PT is active during the early stance and terminal swing phases of gait, particularly during deceleration and pivoting. It contracts eccentrically to control tibial rotation and to stabilize the lateral meniscus [18]. These dynamic contributions are often unaccounted for in traditional surgical planning, yet may significantly affect outcomes in athletic populations.

In high demand sports, such as skiing, football, and martial arts, the PT may sustain microtrauma or overuse injury, particularly when tibial rotation forces are excessive. Understanding its role in dynamic stabilization supports the rationale for rehabilitation strategies that emphasize proprioception, rotational control, and popliteus specific strengthening [19].

### 3.5. Implications for Biomechanical Modeling and Surgery

Modern finite element models of the knee have incorporated the PT as a separate stabilizing unit, improving the predictive accuracy for rotational kinematics and joint contact pressures [3]. Such models underscore the necessity of restoring the PT’s anatomical and functional trajectory in surgical reconstructions to reestablish physiological joint mechanics.

Furthermore, surgical techniques, such as anatomic PLC reconstruction, should account for the PT’s native path and interaction with adjacent structures. Reconstructing only the FCL and the PFL without consideration of the PT may yield suboptimal rotational control [1,3].

## 4. Clinical Significance of the Popliteus Tendon

The PT plays a vital, yet often underestimated, role in the stabilizing function of the PLC of the knee joint. Its primary function is to resist external tibial rotation and to assist in limiting posterior translation, particularly when the knee is in a flexed position [1]. The PT also acts as an “unlocking” mechanism during the initiation of knee flexion by internally rotating the tibia during early flexion [15].

The clinical relevance of this structure is especially evident in rotational injuries of the knee, where the PT may be damaged either in isolation or as part of a combined injury involving the FCL, the lateral meniscus, or the PCL [6]. Failure to diagnose or address a PT injury can result in persistent posterolateral instability, impaired rotational control, and eventual failure of the ACL reconstruction [18,19].

Biomechanical studies confirm that transection of the PT significantly increases external tibial rotation and posterior displacement under load, particularly in flexed positions [1,27]. Thus, the integrity of the PT is essential to maintaining the functional stability of the knee, and it plays a crucial role in ligamentous reconstruction and revision procedures [3]. Biomechanical studies have confirmed that transection of the PT significantly increases the external tibial rotation and the posterior displacement under load, particularly in flexed positions [1,27]. Thus, the integrity of the PT is essential for maintaining functional stability of the knee, and it plays a crucial role in ligamentous reconstruction and revision procedures [3].

Recent advancements have further clarified the PT’s mechanical contributions. Inverse dynamic modeling has demonstrated that the PT provides critical resistance to external tibial rotation, with peak activity occurring between 30° and 60° of knee flexion during deceleration and pivot tasks [8]. Notably, the tendon’s oblique orientation enables it to counteract varus stress and posterior tibial translation by forming a stabilizing vector that opposes multiplanar loads. In vivo neurophysiological evidence confirms that the PT integrates into the sensorimotor feedback loop, actively contributing to rotational control and proprioceptive regulation during stance and directional changes [10].

This underscores the need to preserve or restore PT function in both acute and chronic posterolateral instability, especially in high-demand populations.

A key clinical challenge is the considerable anatomical variability of the PT. The most recent classification by Olewnik et al. [11] identifies four major types of PT morphology, along with several subtypes, based on the presence and insertion of accessory bands. These bands may attach to the lateral meniscus, the FCL, the posterior joint capsule, the oblique popliteal ligament, or the posterior meniscofemoral ligament [11].

The presence of these accessory bands can lead to misinterpretation in MRI or during arthroscopy. Bands attaching to the lateral meniscus (Types IIc, IVc, IVd, IVe) may be mistaken for fibrous adhesions or pathological structures [1,6]. Such misinterpretation may result in their resection, which could compromise the biomechanics of the PLC.

Another challenge is the presence of two distinct tendinous structures (Types III and IV), which may be mistaken for ruptured or hypertrophic tissue, potentially leading to incomplete repair or unnecessary removal [11]. These configurations are particularly important in reconstructive and revision surgeries, where accurate reestablishment of the native tendon anatomy is essential for restoring knee stability.

During PLC reconstruction or ACL revision, careful identification of the PT is recommended, and preservation of its integrity is advised where possible [1]. Modern arthroscopic techniques allow for visualization of the tendon, though their success depends on the surgeon’s awareness of the potential anatomical variants. Preoperative imaging using MRI or high resolution ultrasound can aid in identifying these configurations [3,11].

Clinical reports have indicated that patients with partial or isolated PT injury may experience subjective knee instability, or “giving way”, during activities involving rotation or uneven terrain, despite intact cruciate and collateral ligaments [6]. Therefore, there is a growing consensus for incorporating a PT evaluation into standard post-injury diagnostic algorithms [19].

In practice, knowledge of the PT’s morphological variability can enhance the diagnostic accuracy of arthroscopy, reduce iatrogenic risk, and improve surgical outcomes in ligament reconstructions. Incorporating anatomical classifications, such as the one proposed by Olewnik et al. [11], can support clinical decision making and promote a more individualized surgical approach.

Clinical experience also highlights specific cases where failure to recognize anatomical variants led to suboptimal outcomes. For instance, in a patient undergoing arthroscopy for presumed lateral meniscal tear, a Type IIc variant with an accessory band attaching to the lateral meniscus was resected, resulting in postoperative posterolateral laxity. In another case, a Type IVe configuration with dual insertions to the meniscus was misinterpreted as synovial fibrosis, leading to unnecessary debridement and chronic instability. Awareness of these variants is thus essential during both diagnostic and surgical procedures.

## 5. Diagnosis and Imaging of the Popliteus Tendon

The PT plays a central role in the stabilization of the PLC of the knee. Due to its deep anatomical position and high morphological variability, accurate evaluation of the PT poses a significant diagnostic challenge, particularly in cases of rotational instability [1,19]. A thorough assessment of the tendon is essential for the appropriate diagnosis and management of injuries affecting this region.

### 5.1. Clinical Evaluation

The clinical diagnosis of PT injuries relies on functional stability tests, such as the dial test, the posterolateral drawer test, and the external rotation recurvatum test, which are used to detect posterolateral rotational instability [6]. However, isolated PT injuries may yield inconclusive or subtle findings with these assessments. Patients often report a sense of “instability”, or “giving way”, during pivoting movements or ambulation on uneven terrain [18].

Due to its deep location beneath the gastrocnemius and semimembranosus muscles, the PT is difficult to palpate directly, making imaging the mainstay of diagnostic evaluation [2].

### 5.2. Magnetic Resonance Imaging (MRI)

**Figure 1:**
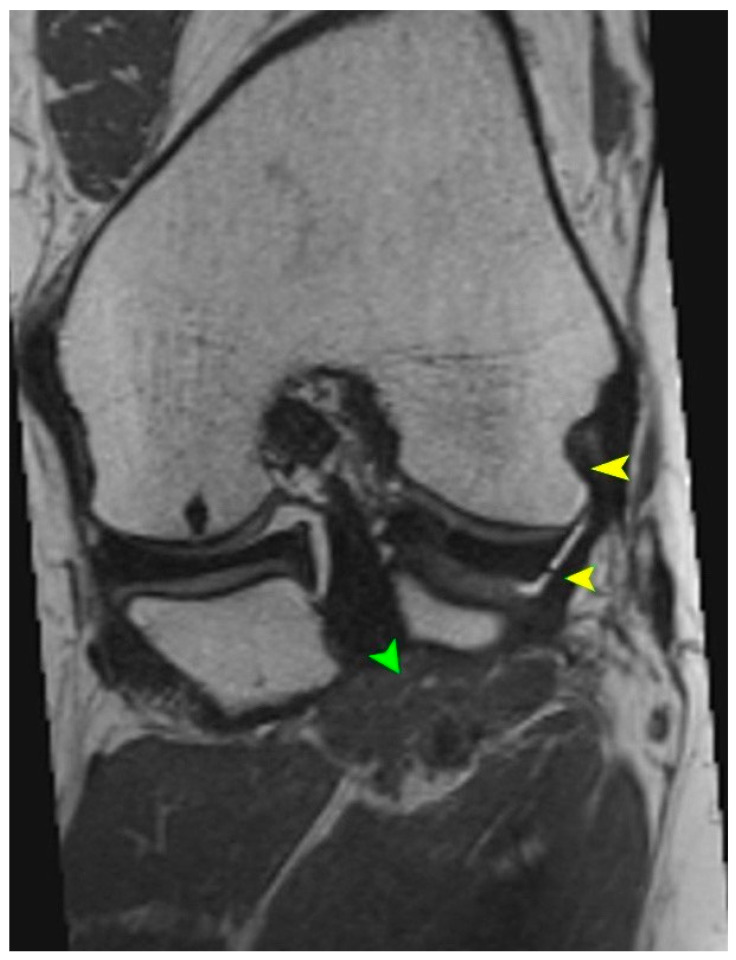
T2 sequence showing the tendon and multiplanar reconstruction. Yellow arrows indicate the tendon of the PM. Green arrows indicate the muscle belly of the PT.

**Figure 2:**
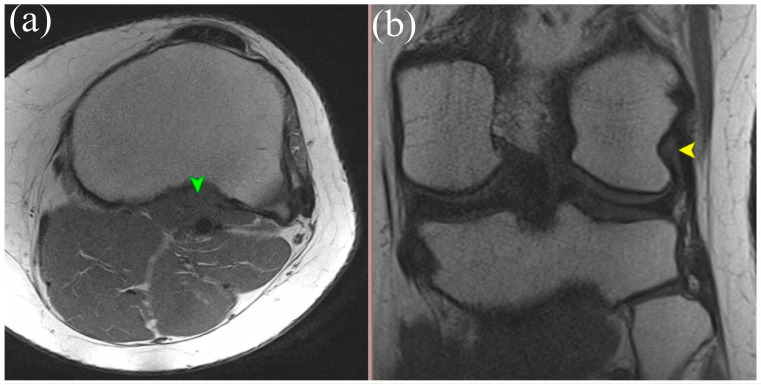
(**a**) Axial T1-weighted MRI showing the muscle belly of the popliteus muscle (green arrowhead). (**b**) Coronal T2-weighted MRI illustrating the tendinous portion of the popliteus (yellow arrowhead). Both panels demonstrate anatomical relationships of the popliteus tendon complex relevant for imaging-based identification.

MRI is the gold standard for evaluating soft tissue structures in the knee, including the PT. Optimal visualization of the tendon is achieved using T1 and T2 weighted sequences (Figure 1 and Figure 2) with fat saturation in axial, coronal, and sagittal planes. The PT appears as a low signal intensity structure running obliquely from the lateral femoral condyle to the posteromedial tibia [1,15].

Particular attention must be given to morphological variations. The presence of bifid tendons or accessory bands (Types II–IV according to Olewnik’s classification) can be mistaken for tendon ruptures, scar tissue, or pathological adhesions [11]. Recognition of these variants is essential to avoid misdiagnosis.

In acute traumatic cases, key findings include hyperintensity within the tendon on T2 weighted images, peritendinous fluid, discontinuity of tendon fibers, and surrounding soft tissue edema [1]. MRI can also reveal the secondary signs of PT injury, such as displacement of the lateral meniscus, stretching of the fibular collateral ligament, and abnormal tibial rotation [15]. Studies have shown that awareness and identification of the anatomical variability of the PT during MRI interpretation of acute knee injuries can improve diagnostic sensitivity and specificity by 15–20% [6].

While MRI provides detailed visualization of the PT and its insertion, it may not always detect fine accessory structures, especially when they are obliquely oriented or overlap with surrounding soft tissue planes [30]. By contrast, high-resolution dynamic ultrasound has been shown to detect accessory bands with greater sensitivity, especially those inserting into the lateral meniscus or fibular collateral ligament [11,31].

Recent comparative studies have suggested that an MRI achieves higher specificity (~95%) for full-thickness tears and deep tissue involvement, while a dynamic ultrasound offers superior sensitivity (~88–92%) for variant morphologies and dynamic instability patterns [30]. When used together, these imaging modalities provide complementary insights, enhancing diagnostic accuracy in both acute and chronic posterolateral pathology.

### 5.3. High-Resolution Ultrasound (US)

**Figure 3:**
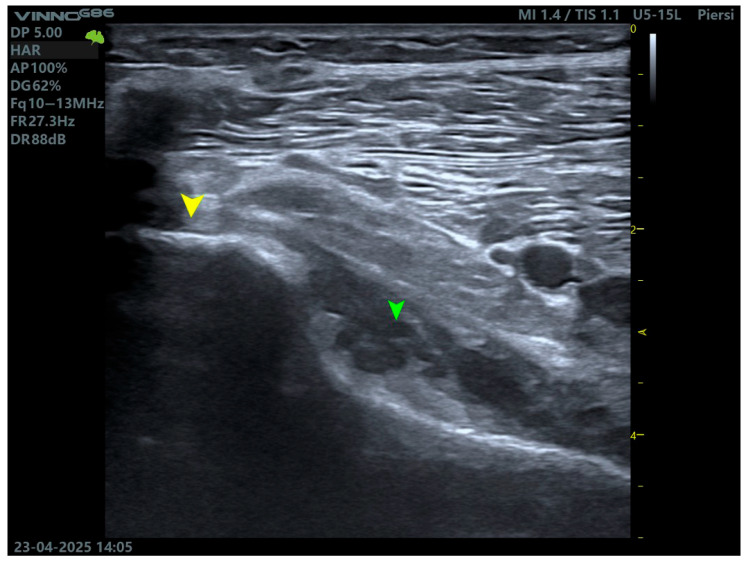
Ultrasound imaging of the popliteus muscle. The yellow arrow indicates the tendon, while the green arrow indicates the muscle belly.

High frequency ultrasonography (≥12 MHz) is gaining relevance as a rapid, dynamic, and non-invasive tool for PT assessment, particularly in outpatient or sports medicine settings [18]. Scanning is performed with the patient in the prone position or with the knee flexed to 30–45°, while applying internal and external tibial rotation. This facilitates the dynamic observation of tendon gliding relative to the adjacent structures and detection of the accessory bands—Figure 3.

An ultrasound enables the evaluation of tendon echotexture, integrity, calcifications, enthesopathy, and peritendinous fluid collections. It is especially useful in the assessment of lateral knee pain following trauma, particularly in cases where MRI is unavailable or contraindicated (e.g., due to metallic implants) [2].

### 5.4. Diagnostic Arthroscopy

In ambiguous cases, diagnostic arthroscopy serves as the most definitive modality, allowing for direct visualization of the PT’s femoral insertion, trajectory, and the presence of accessory structures [3]. Arthroscopic access is typically obtained via the anterolateral portal with knee rotation, and enhanced by the use of posterolateral portals.

Reports have described the arthroscopic identification of bifid PTs (Types III and IV) and the ability to distinguish normal anatomical variants from adhesions. This is particularly critical during revision surgery or PLC reconstruction, where precise anatomical knowledge directly affects outcomes [6].

### 5.5. Preoperative Imaging and Surgical Planning

Preoperative identification of PT morphology is critical for safe and effective surgical planning. Awareness of accessory bands inserting onto the lateral meniscus or the FCL can help avoid iatrogenic injury, particularly during meniscal repair or PLC reconstruction [7].

Moreover, in procedures such as PLC reconstruction or ACL revision, preoperative imaging allows the surgeon to anticipate the anatomical variant and to select appropriate tunnel placements, graft configurations, and suture anchor positions [3].

Incorporating a PT evaluation into routine imaging protocols, particularly in patients with unexplained rotational instability or lateral knee pain, may lead to more accurate diagnoses, tailored treatment, and improved outcomes [1].

A variety of imaging modalities are employed to assess the morphology and pathology of the PT, each with unique advantages and limitations depending on the clinical context. MRI remains the gold standard for a non-invasive evaluation due to its superior soft tissue resolution. Ultrasound, although operator dependent, allows for dynamic and cost-effective visualization, particularly valuable in outpatient and sports medicine settings. By contrast, diagnostic arthroscopy, while invasive, offers direct visualization and therapeutic potential. A comparative summary of these modalities, including their diagnostic sensitivity, benefits, and constraints, is presented in Table 2.

## 6. Surgical Implications of Popliteus Tendon Morphological Variability

The morphological variability of the PT carries substantial clinical and surgical significance, particularly for procedures involving the PLC of the knee, ACL revision surgeries, and both diagnostic and interventional arthroscopy. A detailed understanding of these anatomical variants can directly impact surgical technique selection, precision in anatomical reconstruction, and reduction in iatrogenic complications [1,11].

### 6.1. Topographical Considerations and Surgical Exposure Planning

The PT originates from the lateral femoral condyle and courses obliquely to the posterior medial aspect of the tibia, running deep to the FCL and the posterior joint capsule. It lies in close proximity to other key PLC structures, including the FCL, the PFL, the lateral meniscus, and the lateral head of the gastrocnemius. Accurate knowledge of this regional anatomy is essential for safe dissection and exposure during open surgery [1,19].

Failure to correctly identify the PT intraoperatively may result in its accidental transection or misidentification as a torn ligament fragment, particularly when accessory bands are present. Such errors can compromise posterolateral stability and alter graft biomechanics [15].

### 6.2. Posterolateral Corner Reconstructions

As a dynamic stabilizer of the PLC, the PT plays a critical biomechanical role. In posterolateral reconstructions, particularly using the LaPrade technique or its variations, the precise anatomical replication of the femoral and tibial insertions of the PT is necessary. Morphological anomalies, such as bifid tendons or accessory fascicles inserting into the FCL, the lateral meniscus, or the posterior capsule, can lead to incorrect tunnel placement or graft misalignment, reducing reconstruction efficacy [3,17].

Biomechanical studies have shown that failure to restore PT function may lead to increased stress on ACL grafts and a higher risk of graft failure, even if the FCL and the PFL are reconstructed appropriately [2].

Morphological variability of the PT may necessitate significant modifications in graft selection and tunnel orientation. For example, in the presence of a bifid tendon (Type III or IV), the native anatomical trajectory may differ from standard expectations, requiring surgeons to adapt the femoral or tibial tunnel positioning to avoid graft malalignment.

Moreover, accessory bands attaching to the FCL or the lateral meniscus (e.g., Types IIb, IIc, IVb, IVc) can obscure surgical landmarks and interfere with the graft passage or fixation. If unrecognized, these anatomical features may lead to iatrogenic damage or suboptimal restoration of rotational stability.

Therefore, detailed preoperative imaging and intraoperative mapping of the PT variants should guide decisions on graft type, tunnel angulation, and fixation strategy to optimize reconstruction outcomes and to prevent failure in high-grade PLC reconstructions [7,11].

### 6.3. ACL Reconstruction and Rotational Instability

Variations in PT morphology are equally relevant during primary and revision ACL reconstruction. In patients with residual rotational laxity or a high grade pivot–shift, unrecognized posterolateral deficiency, especially involving the PT, can compromise graft stability. Studies have reported that failure to address this can lead to increased anterior translation and early graft deterioration [18,32].

Intraoperative preservation of PT integrity and the consideration of concurrent PLC reconstruction are recommended for patients demonstrating ≥ 10° of external rotation asymmetry (positive dial test), suggestive of a combined ACL/PLC injury [1].

### 6.4. Arthroscopy and Risk of Iatrogenic Injury

In diagnostic arthroscopy, the PT may be misinterpreted as fibrotic tissue, a synovial fold, or pathological adhesion. This is especially true in cases with accessory fascicles attaching to the lateral meniscus or capsule (e.g., Types IIc, IVc, or IVe in Olewnik’s classification). Resection of these native stabilizing bands due to misidentification can lead to iatrogenic instability or meniscal dysfunction [11].

During arthroscopic procedures involving the lateral gutter or posterolateral compartment, such as meniscal repair or lateral meniscectomy, unrecognized fascicles of the PT may be injured if mistaken for abnormal tissue. Preoperative awareness and intraoperative identification of these variants are crucial for anatomical preservation and functional outcomes [3].

#### Clinical Pitfalls in Arthroscopic Evaluation of the Popliteus Tendon

- Accessory bands (Types IIc, IVc, IVe) may mimic lateral meniscal tears or fibrotic adhesions.
- Dual tendons (Types III, IV) can be misinterpreted as torn or duplicated structures.
- Lack of awareness of PT variants may lead to unnecessary debridement or incomplete reconstruction.
- Misidentification during revision procedures can result in persistent posterolateral instability.

### 6.5. Open Surgery and Identification of Variant Attachments

Cadaveric studies have demonstrated that the PT can insert into multiple anatomical landmarks, including the lateral femoral condyle, the lateral meniscus, the fibular head, the oblique popliteal ligament, and the meniscofemoral ligaments. In multiligament knee injuries, failure to correctly identify these variants may result in inadequate or incomplete reconstruction of the stabilizing structures [1,7].

Notably, Types III and IV PT configurations, characterized by dual tendinous components, require particular caution during posterolateral dissection to avoid misinterpretation as scar tissue or duplication artifact [15].

### 6.6. Surgical Recommendations

To address the anatomical complexity and variability of the PT, the following best practices are recommended:

- Preoperative MRI evaluation of PT morphology should be standard, especially for revision ACL or suspected PLC injuries. Specific attention should be paid to the number of tendinous structures, their orientation, and associated fascial connections [1,11,33].
- Use of intraoperative ultrasound or dynamic palpation may assist in confirming the presence of accessory bands, especially for open reconstructions where visualization is limited [18].
- Intraoperative mapping of the PT and its insertions guided by classifications such as that proposed by Olewnik et al. [11] can prevent inadvertent damage to stabilizing fascicles.
- Preservation of native PT tissue should be prioritized unless the pathology dictates otherwise; reconstruction of its stabilizing bands should be considered in high-grade PLC injuries [3].
- In cases of posterolateral reconstruction failure or unexplained lateral instability, consider a re-evaluation of unrecognized accessory bands or misinterpreted anatomy as potential contributors [6,19].

## 7. The Role of the Popliteus Tendon in Sports Orthopedics

The PT plays a critical, though often underappreciated, role in the domain of sports orthopedics. It acts as a dynamic stabilizer of the PLC of the knee and is essential in controlling tibial rotation, maintaining lateral meniscal stability, and initiating knee flexion functions that are vital in high-performance and pivoting sports [1]. Despite its biomechanical relevance, PT injuries in athletes remain frequently misdiagnosed or overlooked [32].

### 7.1. Mechanisms of Injury in Athletes

Athletes are particularly susceptible to popliteus-related pathology due to the high demand for multiplanar stability during sport-specific movements. The PT may be injured as follows:

- Acutely, due to direct trauma or forced hyperextension with external tibial rotation (e.g., skiing, football).
- Chronically, via repetitive microtrauma from excessive torsional stress, especially in runners, martial artists, and athletes involved in cutting or pivoting sports [3].

Isolated PT tears are rare but have been reported in contact sports and alpine skiing, often presenting with localized posterolateral knee pain and rotational instability. Due to the deep anatomical location and overlapping symptoms with lateral meniscal pathology, these injuries are frequently misattributed or underdiagnosed [3].

### 7.2. Popliteus Tendon Syndrome and Overuse Conditions

In athletes, overuse of the PT may result in popliteus tendonitis or popliteus tendinopathy, characterized by localized tenderness just posterior to the fibular head, pain during downhill running or knee flexion under load, and occasional snapping sensations [18]. This condition may coexist with other forms of posterolateral overuse syndromes, such as iliotibial band friction syndrome.

In high-level runners, repeated tibial internal rotation may result in eccentric overloading of the PT, predisposing it to tendinosis or tenosynovitis. In such cases, ultrasound guided peritendinous injections or eccentric rehabilitation protocols have shown efficacy in symptom resolution [6].

### 7.3. Popliteomeniscal Fascicle Injury in Pivoting Sports

Another unique pathology linked to the PT in athletes is injury to the popliteomeniscal fascicles fibrous extensions from the PT to the posterior horn of the lateral meniscus. These fascicles help stabilize the meniscus during knee flexion and internal rotation [1,18]

Injuries to the popliteomeniscal fascicles are typically seen in soccer players and basketball athletes, and present with symptoms of lateral meniscal instability, locking, or pain during deep flexion. These are often difficult to detect on standard MRI but can be visualized via dynamic arthroscopy [6]. Failure to recognize and repair these structures can result in persistent posterolateral pain or failed meniscal repair [15].

### 7.4. Implications for Surgical Decision-Making in Athletes

In athletes undergoing ACL reconstruction, concurrent injuries to the PLC particularly involving the PT must be identified and addressed to prevent residual instability. Persistent pivot–shift or lateral laxity despite an anatomically placed ACL graft may point to an unrecognized PT injury [3,19].

In elite athletes, isolated PT repair or debridement has been reported to yield excellent functional outcomes when properly diagnosed. Return-to-sport timelines are generally shorter for tendinopathies compared to structural ligament injuries, particularly when managed with sport-specific rehabilitation and neuromuscular re-education [18,34].

For high-demand individuals with chronic posterolateral instability, anatomic reconstruction of the PT as part of a broader PLC reconstruction has been shown to restore rotational control and to enable return to elite competition levels [1,11].

### 7.5. Rehabilitation in the Athletic Population

Athletes recovering from PT related injury should follow a progressive protocol that includes the following:

- Restoration of full range of motion,
- Early proprioceptive training,
- Emphasis on controlling rotational movements during weight-bearing activities,
- Integration of sport-specific drills targeting multiplanar stability [18,23,35].

Rehabilitation should also include eccentric and isometric loading of the posterior chain, including the popliteus, especially for runners and jumping athletes [6,35]. Functional bracing may be temporarily indicated in high-risk return-to-play scenarios [34].

## 8. Rehabilitation Following Popliteus Tendon Injury or Reconstruction

Rehabilitation after injury or surgical intervention involving the PT requires an individualized, structured approach that accounts for the tendon’s unique biomechanical role within the PLC of the knee. Given its responsibility for controlling external tibial rotation, stabilizing the lateral meniscus, and supporting dynamic movements, such as pivoting and cutting, restoring PT function is essential for both recreational and elite level patients [1,6].

### 8.1. Phase-Based Rehabilitation Strategy

Rehabilitation should be staged according to the healing constraints and functional goals as follows:

- Initial Phase (Weeks 0–2): Focus on minimizing inflammation and pain, while preserving range of motion. Passive mobility exercises are initiated, while weight-bearing may be limited or assisted depending on the surgical method (e.g., PT repair or PLC reconstruction). Early quadriceps and gluteus activation are essential [23,36].
- Early Strengthening (Weeks 2–6): Controlled load introduction begins with closed kinetic chain exercises and proprioceptive retraining. Modalities like low-resistance cycling and neuromuscular re-education help maintain joint function and muscular coordination [18,23,36].
- Functional Phase (Weeks 6–12): The emphasis shifts toward restoring strength, enhancing dynamic control, and improving rotation-specific stability. Activities include single leg exercises, lateral movements, and neuromuscular drills to challenge tibial rotational control [18,23,36].
- Advanced Phase (Weeks 12+): Athletes and active individuals progress to high-level strength and plyometric work, including cutting, change of direction drills, and return-to-sport simulations. Objective testing (e.g., hop tests, Y-balance) ensures readiness and limb symmetry [18,23,36].

Return to full athletic activity typically occurs no earlier than 5–6 months postoperatively, with decisions guided by functional milestones rather than time alone [18,23,34,36].

### 8.2. Tendinopathy Management

In cases of chronic tendinopathy or low-grade partial tears, conservative treatment may be effective. A regimen of progressive eccentric loading, isometric exercises, and kinetic chain strengthening, particularly targeting the hamstrings and gastrocnemius, has been associated with favorable outcomes [1,23]. Manual therapy and soft-tissue mobilization may assist in pain modulation and restoring local tissue mobility [36].

### 8.3. Special Considerations

- Following PLC reconstructions, restrictions on tibial rotation and resisted external rotation may be necessary for up to 10 weeks to protect the healing structures [3,19].
- Adjunct modalities, such as blood flow restriction therapy or neuromuscular electrical stimulation, can support strength maintenance and neuromuscular activation when traditional loading is contraindicated [18,35].
- Imaging-guided monitoring, particularly with ultrasound, may assist in tracking tendon healing and optimizing load progression, especially in cases of chronic overuse [30,31].

A structured, phase-based rehabilitation protocol tailored to popliteus tendon pathology can enhance clinical outcomes and can reduce the time to a safe return to play. Table 3 outlines the key goals, interventions, and progression criteria for each rehabilitation stage.

## 9. Summary

Rehabilitation of the PT should be adapted to the nature of the injury and surgical repair. Priority must be given to restoring neuromuscular control, proprioceptive function, and dynamic stability, especially rotational control, to prevent residual instability or reinjury. Return-to-sport protocols should emphasize movement quality, not just strength, and be guided by functional criteria validated in both the clinical and the sports performance literature.

## 10. Biomarkers and Laboratory Correlates in Popliteus Tendon-Associated Pathology

Although no biomarker is currently specific to popliteus tendon (PT) pathology, growing evidence supports the utility of synovial and serum biomarkers in evaluating soft tissue injury within the knee joint—particularly in cases involving posterolateral corner (PLC) trauma and rotational instability. Studies have demonstrated that elevated levels of C-reactive protein (CRP) and interleukin-6 (IL-6) are associated with acute joint inflammation and may correlate with the severity of intra-articular soft tissue damage [37].

Further, matrix degradation enzymes, such as matrix metalloproteinases (e.g., MMP-3), have been detected in higher concentrations following acute ligamentous and meniscal injuries, and may be linked to early degenerative changes [20]. In particular, MMP-3 levels rise sharply in response to tissue remodeling, making it a potential adjunctive marker for evaluating structural compromise in PLC- or PT-involved lesions.

Biomarker expression may also offer prognostic information in post-traumatic osteoarthritis following ACL–PLC associated injuries, as shown by the gene-expression and proteomic profiling of both ACL tissue and synovial fluid [38]. These molecular signatures can support the stratification of patients for tailored rehabilitation timelines and surgical decision-making. Additionally, they may assist in distinguishing between mechanical and inflammatory contributors to persistent instability or lateral knee pain [39].

In the context of sports medicine, ongoing monitoring of inflammatory biomarkers during rehabilitation may offer value in guiding return-to-play decisions, particularly in athletic populations recovering from rotational trauma. However, further studies are needed to validate biomarker thresholds and to establish specific reference ranges for PT-associated injuries.

## 11. Conclusions

1. The PT exhibits substantial morphological variability, including differences in the number of fascicles, insertion sites, and the presence of accessory bands inserting into the lateral meniscus, the FCL, or the posterior capsule.
2. The classification proposed by Olewnik et al. (2021) [11] currently represents the most comprehensive anatomical system for describing these variations, and provides a structured approach for both clinical and anatomical assessment.
3. Anatomical variability has direct surgical implications, influencing the planning and success of PLC reconstructions, ACL revisions, and contributing to persistent rotational instability even after anatomically performed procedures.
4. The PT is at risk of misinterpretation during arthroscopy, where it may be confused with fibrotic tissue or pathological adhesions, increasing the risk of inadvertent resection.
5. Advanced imaging (MRI and high-resolution ultrasound) should include targeted assessment of the PT, particularly for patients with lateral or rotational knee instability or for preoperative planning for complex reconstructions.
6. The integration of anatomical classification, high-resolution imaging, and proprioceptive function of the popliteus should become a standard part of interdisciplinary management, involving orthopedic surgeons, radiologists, and physical therapists.
7. The classification of the popliteus tendon should also be integrated into arthroscopic training and preoperative planning tools, including surgical navigation and robotic-assisted systems, to enhance anatomical precision and intraoperative decision-making.

## 12. Future Research Directions

1. Multicenter anatomical and cadaveric studies using high-resolution 3D reconstruction, to validate and to expand the existing classification systems of the PT.
2. Correlative imaging dissection studies (MRI vs. cadaver) to improve the recognition of accessory fascicles and bifid tendon types in clinical diagnostics.
3. Development of arthroscopic and reconstructive surgical protocols that incorporate PT classification, including decision-making algorithms for rotational instability despite intact cruciate ligaments.
4. Integration of PT anatomical data into navigation-assisted and robotic surgical systems, especially in the context of personalized tunnel placement and ligament balancing procedures.
5. Design of dynamic functional tests for popliteus activity, enabling an evaluation of tendon function without relying solely on imaging—potentially applicable in orthopedic or rehabilitation clinics. Such tests may focus on the control of tibial external rotation under load, posterior translation during knee flexion, and response to multiplanar instability challenges during stance and directional changes.
6. Inclusion of the PT anatomy as an evaluated variable in clinical studies assessing the outcomes of PLC reconstruction and ACL revision surgery—possibly as a prognostic factor.
7. Investigation of popliteus-specific rehabilitation strategies and their effect on return-to-sport metrics, particularly in pivoting and high-demand athletic populations.

The following clinical scenarios in Table 4 outline the specific recommendations for diagnosis, imaging, rehabilitation, and surgical planning in cases where popliteus tendon pathology or variability may influence outcomes.

## Figures and Tables

**Table 1:** Anatomical Classification of the Popliteus Tendon (Olewnik et al.) with Clinical and Surgical Relevance.

Type	Description	Prevalence	Clinical and Surgical Implications
Type I	Single tendon attached to the proximal half of the popliteal sulcus	46 limbs (34.3%)	Most common type; typically visualized clearly on imaging and during surgery; considered the anatomical “baseline”.
Type II	Single tendon with accessory bands; main tendon as in Type I	41 limbs (30.6%)	May be mistaken for fibrous adhesions or scarring; accessory bands can be confused with pathological structures in arthroscopy.
IIa	One accessory band attaching to the oblique popliteal ligament	17 limbs	Potential misdiagnosis as capsular thickening on MRI.
IIb	One accessory band attaching to the fibular collateral ligament	12 limbs	Can complicate interpretation of lateral stabilizer continuity.
IIc	One accessory band attaching to lateral part of the lateral meniscus	6 limbs	May mimic meniscocapsular lesions; should not be resected.
IId	Two bands: one to the posterior capsule, one to the oblique popliteal ligament	4 limbs	Increases surgical complexity; may require modification of tunnel orientation in PLC reconstructions.
IIe	Three bands: to the FCL and posterior meniscofemoral ligament (2)	2 limbs	Rare; risk of neurovascular entrapment if unrecognized.
Type III	Two separate tendons inserting into the popliteal sulcus	21 limbs (15.7%)	Can be confused with a split tear or a bifid tendon; critical to differentiate in preoperative imaging.
Type IV	Two tendons as in Type III, with additional accessory bands	26 limbs (19.4%)	Complex variant; increases the chance of diagnostic and surgical misinterpretation.
IVa	One accessory band to the oblique popliteal ligament	5 limbs	May interfere with posterior capsular release.
IVb	Two bands: to the FCL and posterior capsule	4 limbs	Mimics scarring or chronic trauma-related fibrosis.
IVc	Bands to the FCL and lateral meniscus	7 limbs	Misinterpretable as meniscotibial injury; affects lateral meniscus mobility.
IVd	Bands to the lateral meniscus and the posterior capsule	6 limbs	Requires care during meniscal repair; risk of unintended band resection.
IVe	Bands to the medial and lateral portions of the lateral meniscus	4 limbs	Unusual configuration; risk of overconstraint if reconstructed improperly.

**Table 2:** Comparative Imaging Modalities for Popliteus Tendon Evaluation.

Modality	Sensitivity for PT	Advantages	Limitations
MRI	90–95%	High resolution, detailed soft tissue view	Expensive, limited access in some settings, less useful for chronic injuries
Ultrasound	70–85%	Dynamic assessment, portable, cost effective	Operator dependent, reduced depth penetration
Arthroscopy	100% (direct view)	Gold standard, diagnostic + therapeutic	Invasive, requires anesthesia

Sensitivity values based on the findings from Smith et al. [31]; Chang et al. [30]; Feipel et al. [15]; Geeslin and LaPrade [6]; LaPrade et al. [1].

**Table 3:** Phase-Based Rehabilitation Protocol for Popliteus Tendon Injuries.

Phase	Goals	Interventions	Criteria for Progression
Phase I: Acute (0–2 weeks)	Reduce pain and inflammation; protect healing structures; restore passive ROM	RICE, protected weight-bearing, gentle passive ROM (0–90°), isometrics of quadriceps and hamstrings	Pain ≤ 3/10, minimal swelling, passive ROM ≥ 90°, able to contract quadriceps without compensation
Phase II: Subacute (2–6 weeks)	Restore full ROM; reintroduce neuromuscular control; begin light strengthening	Active ROM, closed-chain exercises, balance training, resistance band work for hip and core	Full ROM, no gait deviation, single-leg stance ≥ 15 s, pain-free level ground ambulation
Phase III: Functional (6–12 weeks)	Enhance strength, proprioception, and dynamic control; initiate sport-specific drills	Dynamic balance, eccentric loading, lateral movements, plyometrics initiation	Symmetric strength ≥ 80%, good control with dynamic valgus tests, tolerance of plyometrics
Phase IV: Return to Sport (12+ weeks)	Restore high-level function; prevent reinjury; optimize neuromuscular patterns	Agility drills, high-velocity multiplanar movements, sport-specific reintegration, functional testing	Completion of return-to-sport testing (e.g., hop tests ≥ 90% symmetry), clearance by physician and therapist; typical return between 12–20 weeks depending on sport level

**Table 4:** Clinical Recommendations Related to the Popliteus Tendon.

Clinical Scenario	Recommended Clinical Action
Isolated lateral knee pain of unclear origin	Evaluate the popliteus tendon using MRI or high-resolution ultrasound
Positive dial test (>10°) during ACL evaluation	Assess for PLC involvement, including PT status
Persistent pivot–shift after ACL reconstruction	Consider missed posterolateral injury involving the PT
Lateral meniscus pathology seen on imaging or arthroscopy	Differentiate between true meniscal injury and PT accessory bands
PLC reconstruction planning	Map popliteus tendon insertions; anticipate morphological variations (e.g., bifid tendon)
Rehabilitation of rotational instability	Include proprioceptive and rotational control exercises targeting popliteus function
Chronic tendinopathy in athletes	Use eccentric strengthening and image-guided therapy targeting the PT
Posterolateral instability in revision ACL cases	Reconstruct or reinforce PT in combination with PLC repair

## Data Availability

Not applicable.

## Abbreviations

PT	Popliteus Tendon
PLC	Posterolateral Corner
ACL	Anterior Cruciate Ligament
PCL	Posterior Cruciate Ligament
PPM	Popliteus Muscle
FCL	Fibular Collateral Ligament
PFL	Popliteofibular Ligament
